# Co_2_TiO_4_/Reduced Graphene Oxide Nanohybrids for Electrochemical Sensing Applications

**DOI:** 10.3390/nano9111611

**Published:** 2019-11-13

**Authors:** Constanza J. Venegas, Fabiana A. Gutierrez, Marcos Eguílaz, José F. Marco, Nik Reeves-McLaren, Gustavo A. Rivas, Domingo Ruiz-León, Soledad Bollo

**Affiliations:** 1 Redox Processes Research Centre (CiPRex), Facultad de Ciencias Químicas y Farmacéuticas, Universidad de Chile, Sergio Livingstone 1007, Independencia, Santiago 8380492, Chile; constanza.jvenegas@gmail.com; 2 Laboratorio de Fisicoquímica y Electroquímica del estado Sólido, Facultad de Química y Biología, Universidad de Santiago de Chile, Av. Libertador Bernardo O’Higgins n° 3363, Santiago 9160000, Chile; 3 INFIQC, Departamento de Físicoquímica, Facultad de Ciencias Químicas, Universidad Nacional de Córdoba, Ciudad Universitaria, Córdoba 5000, Argentina; fabigutierrez@gmail.com (F.A.G.);; 4 Instituto de Química Física Rocasolano, CSIC, Calle Serrano 119, 28006 Madrid, Spain; jfmarco@iqfr.csic.es; 5 Department of Materials Science and Engineering, University of Sheffield, Sheffield S1 3JD, UK; n.reeves@sheffield.ac.uk; 6 Advanced Center for Chronic Diseases (ACCDiS), Facultad de Ciencias Químicas y Farmacéuticas, Universidad de Chile, Santiago 8380494, Chile

**Keywords:** Hybrid materials, Co_2_TiO_4_, reduced graphene oxide, ex situ synthesis, in situ synthesis, H_2_O_2_ detection, electrochemical sensors

## Abstract

For the first time, the synthesis, characterization, and analytical application for hydrogen peroxide quantification of the hybrid materials of Co_2_TiO_4_ (CTO) and reduced graphene oxide (RGO) is reported, using in situ (CTO/RGO) and ex situ (CTO+RGO) preparations. This synthesis for obtaining nanostructured CTO is based on a one-step hydrothermal synthesis, with new precursors and low temperatures. The morphology, structure, and composition of the synthesized materials were examined using scanning electron microscopy, X-ray diffraction (XRD), neutron powder diffraction (NPD), and X-ray photoelectron spectroscopy (XPS). Rietveld refinements using neutron diffraction data were conducted to determine the cation distributions in CTO. Hybrid materials were also characterized by Brunauer–Emmett–Teller adsorption isotherms, Scanning Electron microscopy, and scanning electrochemical microscopy. From an analytical point of view, we evaluated the electrochemical reduction of hydrogen peroxide on glassy carbon electrodes modified with hybrid materials. The analytical detection of hydrogen peroxide using CTO/RGO showed 11 and 5 times greater sensitivity in the detection of hydrogen peroxide compared with that of pristine CTO and RGO, respectively, and a two-fold increase compared with that of the RGO+CTO modified electrode. These results demonstrate that there is a synergistic effect between CTO and RGO that is more significant when the hybrid is synthetized through in situ methodology.

## 1. Introduction

In the last decade, nanomaterial technology has grown, in particular driven by the special properties of nanomaterials [1,2,3,4,5,6,7,8,9,10,11,12,13,14,15,16,17,18,19,20,21,22,23]. Currently, the mixture of different types of nanomaterials among metals, metal oxides, nanoclays, quantum carbon nanodots, and carbonaceous materials such as graphene, reduced graphene oxide, and nanosilica, has allowed the generation of new hybrid structures, widening their spectrum of applications [3,4]. Thus, the fabrication of hybrid materials via electrocatalysis has improved, especially for the design of new electrochemical sensors and for performing analytical tests [5,6,7]. In particular, some of these advantages, including enhanced surface kinetics and accelerated electrochemical reactions, can be achieved by using new nanomaterials in electrode modifications; furthermore, the addition of carbonaceous nanomaterials enhances the adsorption of the analyte on the electrode surface, helping achieve even quantification [8].

As a component of hybrid architectures, reduced graphene oxide (RGO) is an important carbon-based nanomaterial (CBNM) used for the development of optimized sensors. RGO presents very interesting properties, such as excellent electrical conductivity, high specific surface area, and good mechanical strength, making it possible to obtain a fast and sensitive electrochemical response [9,10,11,12,13]. RGO has been used as a support for nanoparticles, improving the electrocatalytic performance of RGO and avoiding nanoparticle agglomeration [14,15]. The CBNM–inorganic oxide hybrid materials induce an additional electrochemical catalytic ability and may provide further functionalization ability. Many examples of these hybrid materials can be found in the literature, where CBNMs are combined with metal nanomaterials (mainly Au, Ag, Pd, and Pt) [16,17], oxides such as Fe_3_O_4_, MnO_2_, Ni(OH)_2_, ZnO, SnO_2_, Co(OH)_2_, and TiO_2_ [18,19,20,21,22,23], and chalcogenides such as CdS, CdSe, and MoS_2_ [24]. The use of these materials has attracted research efforts due to their considerable potential as enhanced materials for sensing applications. Sensors based on carbon–inorganic oxide composites need (i) the inorganic phase to remain stable in ambient conditions without any structural change, (ii) metal species with more than one oxidation state, and (iii) need to be good conductors of charge carriers (ions and electrons) [25].

In CBNM–inorganic oxide hybrids for sensors, distribution of the inorganic phase and carbon-based material on the surface is important for optimal chemical performance of the as-prepared electrode, so their synthesis must consider several factors. The synthesis methods for these new composite materials are generally classified as ex situ and in situ [26]. The ex situ method involves a mixture of CBNM and previously synthesized nanocrystals, either from the inorganic phase or commercially available in solutions. Although in ex situ methods it is possible to preselect nanostructures with desired functionalities, the generated hybrid material sometimes suffers from non-uniform coverage or low density of the nanostructures on the electrode surface. In contrast, in situ methods generally give better coverage of nanocrystals on surfaces by controlling the nucleation sites on CBNM via surface functionalization. Thus, a continuous film of nanoparticles (NPs) on CBNM surfaces can be obtained. The literature reports show an array of different strategies for preparing oxide nanocomposites, such as sol-gel and solid-state techniques, chemical bath deposition, hydrothermal synthesis, and chemical vapour deposition [23,27,28,29,30,31]. Among these, most methods for the production of nanosized oxides are based on either co-precipitation or hydrothermal methods.

Spinel ferrites have received significant attention in electrochemical sensor applications [21,32,33]. They present interesting physical electronic, optical, and magnetic properties that make them very interesting nanomaterials. Other kinds of oxides that contain cobalt in the structure have also attracted attention, with Co_3_O_4_ being widely studied either alone or as a composite with graphene [34,35]. An interesting study published by Qi et al. [34] highlighted the formation of Co_3_O_4_ nanorod/graphene composites for the detection of a specific DNA sequence.

In recent years, there has been increasing interest in the use of hybrid materials combining inorganic oxides and graphene, specifically for the electrochemical detection of hydrogen peroxide, with studies focusing on Co_2_SnO_4_/RGO [36], Pt-CeO_2_/graphene oxide [37], Ni_x_Co_3−x_N/nitrogen-doped graphene [38], and RGO-SnO_2_ [39]. The rapid and accurate determination of hydrogen peroxide has an important role in the electron transfer process of hundreds of enzymes in biological systems [40] connected with the oxidation of by-products of glutamate, oxalate, cholesterol, D-amino acid, urate, lactate, lysine, and glucose, as well as in various types of industries, such as paper, textile, pharmaceutical, and environmental industries [41].

Considerable research has focused on compounds with spinel-type structures [27,28,29,30,42,43]. Ferrite spinels have been the most studied and have been reported in a large number of publications due to their versatile applications, including catalytic functions [43,44]. However, there are no reports on the synthesis of Co_2_TiO_4_/RGO nanocomposites or their application as electrode materials for electrochemical sensors.

According to the literature, Co_2_TiO_4_ (CTO) can be synthesized using various methods (e.g., co-precipitation [45,46], solid-state [47,48,49] or solvothermal syntheses [50]) that always involve a final high-temperature calcination step. In this report, we discuss the fabrication of CTO at reduced temperatures using a hydrothermal synthesis and new precursors, which allow the formation of single-phase CTO in one step. Only a few applications have been reported for CTO, such as the degradation of water pollutants [50] and as anodes in batteries [51]; however, no applications have been reported in the field of electrochemical sensors.

In this work, we propose two methods for the formation of CTO and RGO hybrids, namely in situ (CTO/RGO) and ex situ (CTO+RGO) methods, to be used for the development of hydrogen peroxide sensors. We determine the catalytic effect of these materials for hydrogen peroxide reduction and their application in the electrochemical detection of hydrogen peroxide (H_2_O_2_) in real samples.

## 2. Experimental Section

### 2.1. Chemical Reagents

Cobalt chloride hexahydrate (Sigma Aldrich, San Luis, MO, USA, 98%), titanium tetrachloride (Sigma Aldrich, San Luis, MO, USA, 99.9%), sodium hydroxide (Merck, Darmstad Germany, 98%), and hydrogen peroxide (Merck, Darmstad Germany, Germany, 30%) were used as received. Reduced Grapheme oxide (RGO) was obtained from Graphenea^®^ (San Sebastian, Spain). Details of the elemental analysis and X-ray fluorescence spectrometric analysis of RGO are included in Supplementary Materials (Tables S1 and S2). Nafion^®^ was purchased from Sigma Aldrich (San Luis, Mo, USA). A pH 12 NaOH solution was used as the supporting electrolyte. All solutions were prepared with ultrapure water (ρ = 18 MΩ cm) from a Millipore Milli-Q system.

### 2.2. Synthesis of the Nanohybrids

Synthesis of CTO: CTO nanoparticles were obtained by a hydrothermal method. Cobalt chloride (CoCl_2_∙6H_2_O) and titanium tetrachloride (TiCl_4_) were dissolved in deionized water to form two transparent solutions. The TiCl_4_ solution was slowly added to an ice cold cobalt chloride solution in a 2:1 target molar ratio of Co/Ti. The NaOH solution was added dropwise to the mixture under magnetic stirring until a concentration of 2.0 M was reached. The reaction was stirred at room temperature for 30 min, and the resulting slurry was then transferred into a 23 mL Teflon-lined stainless steel autoclave. The mixture was exposed to hydrothermal conditions at 250 °C for up to 12 h. The resulting product was collected by centrifugation, washed with deionized water and absolute ethanol several times, and then dried at 80 °C.

Ex situ synthesis of CTO+RGO: RGO was mechanically mixed with the CTO nanoparticles in an agate mortar at a ratio of 80:20.

In situ synthesis of CTO/RGO: The same procedure was used for the synthesis of the CTO, but after mixing all the precursors, RGO was added. The hydrothermal syntheses then proceeded using the same time and temperature conditions.

### 2.3. Modification of Glassy Carbon Electrodes (GCEs) with the Nanohybrid Materials

To modify the GCEs, a portion of the hybrid material (CTO+RGO or CTO/RGO) was dispersed in 1.00 mL of Nafion^®^ (0.2% *v*/*v* in ethanol) by sonication for 30 min. Prior to surface modification, the GCEs were polished with 0.3 and 0.05 µm alumina slurries for 1 min. Immobilization of the hybrid nanomaterials was achieved by drop casting 10 µL of the dispersion onto the GCE, followed by evaporating the solvent at room temperature.

### 2.4. Characterization

Room temperature time-of-flight (ToF) neutron powder diffraction (ND) data were collected on the General Material diffractometer (GEM) in a vanadium canister at the ISIS ((Neutron and Muon Source) facility, Rutherford Appleton Laboratory (RAL), Oxford. Data were collected in the backscattering detector bank (50.07°–74.71°). X-ray diffraction (XRD) data were collected in a PANalytical X’Pert^3^ Powder diffractometer with Cu Kα radiation. Rietveld refinement was performed using the (graphical user interface for GSAS experiment, XPGUI [52] for GSAS (Generalized Structure Analysis System) [53] and both XRD and ND data. Raman measurements were recorded with a WiTec Alpha 300 Raman-AFM using a 532 nm laser.

X-ray photoelectron spectroscopy (XPS) data were recorded with a PHOIBOS 150 hemispherical analyzer (SPECS) under a pressure lower than 2 × 10^−9^ mbar using Al Kα radiation and constant pass energy values of 100 eV and 20 eV for the wide and narrow scans, respectively. The binding energy (BE) scale was referenced to the main C 1s signal (284.6 eV) corresponding to the contamination layer. Atomic ratios were calculated using MultiQuant XPS software [54], with the different spectral areas obtained by peak integration after background subtraction using the Shirley method.

The surface morphology was obtained by scanning electron microscopy (SEM) (TESCAN, Czech Republic, Vega 3 model). Histogram size distribution was calculated using ImageJ software. A compositional study was conducted by analysis of energy dispersive spectroscopy (EDS) using a Bruker probe (model QUANTAX 400a series).

Thermogravimetric analysis was carried out with a Shimadzu DTG-60 instrument in a flowing air atmosphere with an increase of 10 °C min^−1^ in platinum canisters. The reference used was α-alumina.

Scanning electrochemical microscopy (SECM) images were obtained with a CHI900 bipotentiostat (CHInstruments, Dallas, TX, USA) using a 10 µm diameter platinum ultra-micro-electrode (UME) probe. The SECM feedback mode was selected to obtain images of each modified surface using a 5.00 × 10^−4^ M ferrocene methanol (FcOH) solution. The UME and the substrate potentials were held at 0.50 V and 0.10 V, respectively, during the acquisition of the images, the UME scan rate was 10.0 µm s^−1^. The SECM surface plots were depicted by normalizing the current of the UME at the surface (i) with the steady-state current of the UME positioned far from the substrate (i_0_).

Textural properties were obtained from the adsorption–desorption isotherm of N_2_ at 77 K, which was carried out using a Micromeritics 3Flex. The sample was previously degassed for 10 h at 76.9 K under vacuum using a Micromeritic SmartVacPrep. The specific surface area was determined from the adsorption branch in the range of 0.05 ≤ p/p_0_ ≤ 0.25 using the Brunauer–Emmett–Teller (BET) theory [55].

Electrochemical impedance spectroscopy (EIS), cyclic voltammetry, amperometry, and polarization curves were performed with a three-electrode cell. Ag/AgCl, 3.0 M KCl (CH Instrument), and platinum wire were used as the reference electrode, electrolyte, and auxiliary electrode, respectively. The working electrode was a glassy carbon electrode (GCE, CH. Instrument) modified with our hybrid materials. Electrochemical impedance spectroscopy (EIS) measurements were performed with an Autolab PGSTAT 128 N potentiostat (EcoChemie) in a frequency range of 10.000 Hz −0.1 Hz (amplitude: ∼10 mV). The redox probe was 0.010 M hydrogen peroxide prepared in pH 12 NaOH, and the working potential was −0.400 V. The impedance spectra were analysed using Z-view software.

Amperometric and voltammetric experiments were performed using a Palm Sens potentiostat (The Netherlands). The amperometric experiments were conducted in deoxygenated pH 12 NaOH solution at –0.400 V by applying the desired working potential and allowing the transient currents to decay to a steady-state value prior to the addition of 0.10 mM H_2_O_2_, with subsequent current monitoring.

Polarization curves were performed in a Bioanalytical Systems, Inc, BAS® (West Lafayette, IN, USA) Model CV 50 W potentiostat at 0.005 V/s using 1.0 mM hydrogen peroxide solution in deoxygenated pH 12 NaOH solution. The rotating disk speed was maintained at 1600 rpm.

## 3. Results and Discussion

### 3.1. Physicochemical Characterization

**Figure 1:**
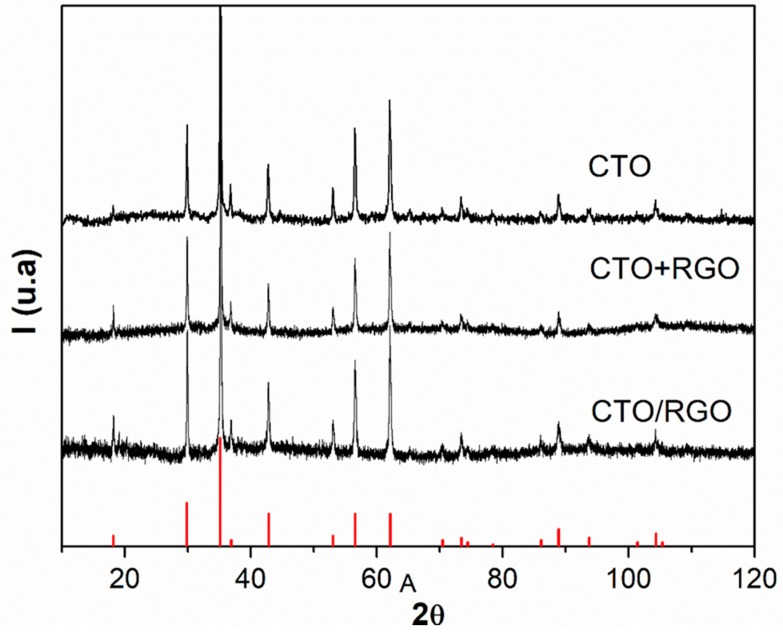
Phase analysis of the diffraction patterns for pristine Co_2_TiO_4_ (CTO), CTO/reduced graphene oxide (RGO) (in situ), and CTO+RGO (ex situ). The red diffraction pattern corresponds to CTO (ICDD PDF # 00-018-0428).

Phase analysis was performed using XRD. Figure 1 shows the diffraction patterns for pristine CTO and both CTO/RGO and CTO+RGO specimens. In all cases, the observed Bragg reflections were indexed against the International Centre for Diffraction Data (ICDD) database entry for Co_2_TiO_4_) (ICDD PDF # 00-018-0428). There were no additional peaks observed that might have been attributable to contaminants or unreacted precursors. The characteristic Bragg peak for RGO (002, a 2θ = 24°) was not observed, suggesting that RGO sheets were not homogeneously dispersed and coated with CTO [56,57], or that its percentage in the hybrids was very low [58,59]. On the other hand, these results confirm that CTO can be obtained using the hydrothermal method with the new precursors, TiCl_4_ and CoCl_2_∙6H_2_O, in a single step. There was no need for high-temperature calcination. The pure CTO was subsequently mixed with RGO to obtain the ex situ hybrid.

**Figure 2:**
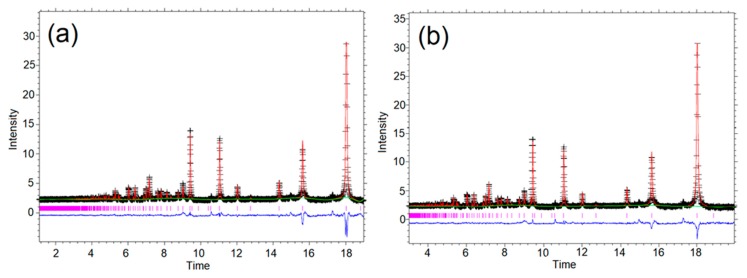
Neutron powder diffraction profile of Co_2_TiO_4_/RGO from Rietveld refinements using GSAS+EXPGUI at room temperature: CTO/RGO (**a**) and CTO+RGO (**b**). The calculated patterns (red) are compared vs. observed data (black +), with the profile difference (observed–calculated) in blue.

To verify the cation distribution within the CTO spinel structure contained in the hybrids, we performed a structural refinement with the Rietveld method using neutron diffraction data. Figure 2 displays the neutron diffraction profiles, which show an inverse spinel-type structure generally described as Co^Td^(Co_0.5_Ti_0.5_)_2_^Oh^O_4_, where cobalt and titanium are expected to be present as Co^2+^ and Ti^4+^. The CTO was refined in the cubic space group, Fd$\bar{3}$m.

The refined parameters are summarized in Table S3. As a starting model, we considered that the Co^2+^ ions to be distributed in both crystallographic, tetrahedral, and octahedral sites, with Ti^4+^ ions distributed only in octahedral sites. The background was refined first, using a shifted Chebyshev function with 16 terms, followed by the lattice parameters, phase fraction scale factors, and the profile parameters, to model peak broadening largely due to crystallite size effects. Finally, the atomic position in order of the scattering factor for metal sites and U_iso_ for oxygen were refined. The process was repeated to convergence, until there were negligible shifts in refined variables. From the occupations observed, it was concluded that the cationic distribution for the CTO in CTO/RGO is (Co_0.98_Ti_0.02_)_Td_(Co_0.96_Ti_1.04_)_Oh_O_4_ and in CTO+RGO is (Co_0.96_Ti_0.04_)_Td_(Co_0.98_Ti_1.02_)_Oh_O_4._ These results indicate that the tetrahedral sites are not fully occupied by Co^2+^ ions since there is a small amount of Ti^4+^, unlike the work reported by S. Thota et al., where CTO was synthesized by a solid-state reaction, with Co^2+^ and Co^3+^ in both crystallographic sites and Ti^3+^ in the octahedral site [48,60]. These differences in the distribution of the cations in the crystallographic sites can be attributed to the method of synthesis and the precursors used, suggesting that the lower reaction temperature possibly means there is insufficient energy to permit full ordering or mixing of the cations, and thus some inhomogeneity persists.

On the other hand, for CTO/RGO and CTO+RGO, similar cell parameter values were calculated from the Rietveld refinements, with a = 8.4519(1) Å and a = 8.4554(1) Å, respectively. The refined values of the lattice parameters were somewhat larger than previously reported for CTO (a = 8.440 Å, ICDD PDF # 00-018-0428), probably due to the formation of a Co_2-x_Ti_1+x_O_4_ solid solution with a small Ti excess [51].

The presence of RGO in the hybrid CTO/RGO was determined by Raman spectroscopy. Figure S1 shows the D band at 1345 cm^−1^ and the G band at 1593 cm^−1^, which is characteristic of carbonaceous materials [61]. The integrated intensity ratio (I_D_/I_G_) of the D and G bands of CTO/RGO, widely used to characterize the degree of defects in graphitic materials [62], was 2.07. In relation to this, the I_D_/I_G_ determined for the RGO present in the hybrids was consistent with those previously reported [61], demonstrating a high degree of defects in the graphitic structure.

**Figure 3:**
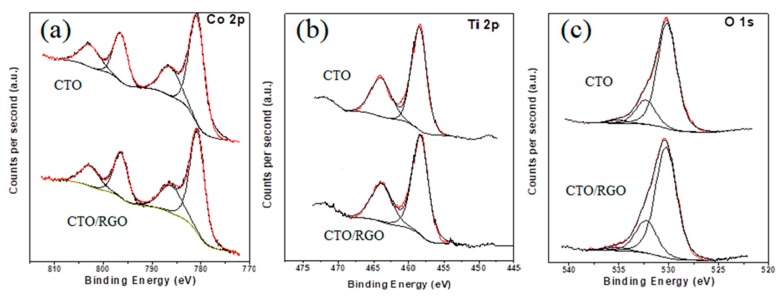
Co 2p (**a**), Ti 2p (**b**), and O 1s spectra (**c**) X-ray photoelectron spectroscopy (XPS) spectra for CTO and CTO/RGO materials.

The chemical compositions of the materials and the valence states of the cations were studied by XPS. The wide scan spectra (not shown) recorded for CTO and CTO/RGO showed only signals corresponding to C, O, Ti, and Co, without contributions from other elements, indicating that the compounds were free from contaminants. The high-resolution Co 2p, Ti 2p, and O 1s spectra recorded for both materials were virtually identical, both in terms of the binding energies of the main spectral features and the shape of the various spectral lines, as observed in Figure 3a–c, respectively.

The Co 2p spectra were composed of a relatively narrow spin–orbit doublet (Binding Energy (BE) Co 2p_3/2_ = 780.7 eV; BE Co 2p_1/2_ = 796.4 eV), accompanied by a strong.“shake-up” satellite structure above the main photoemission lines (786.1 eV and 802.8 eV) (Figure 3a). These binding energy values and the occurrence of the intense satellite features were fully compatible with the presence of Co^2+^ [63]. Although the fairly intense satellites suggested that the materials exclusively contained Co^2+^, we explored the possible existence of a Co^3+^ contribution, which had been recently observed elsewhere [64]. It is known that oxides with spinel-related structures contain Co^3+^ that can be characterized by a spin–orbit doublet with a binding energy of the main Co 2p_3/2_ core level of 779.6 eV [63]. Including such a contribution in the spectra presented in Figure 3a proved difficult, with the fit always either giving a negligible intensity for the Co^3+^ doublet or unrealistically narrow line widths. Therefore, we can confidently conclude that the oxides prepared in this work contain only Co^2+^.

The Ti 2p spectra recorded for both materials (Figure 3b) showed only a spin–orbit doublet with BE Ti 2p_3/2_ = 458.4 eV and BE Ti 2p_1/2_ = 463.8 eV. These binding energy values are characteristic of Ti^4+^ [65,66,67]. In principle, as we have demonstrated in previous papers [66,67,68], Ti^4+^ can be easily distinguished from Ti^3+^ in a Ti 2p spectrum, and their relative contributions can be determined without problems. In the case of the concomitant presence of Ti^4+^ and Ti^3+^, the Ti 2p lines become broad and asymmetric, since Ti^3+^ is usually characterized by a spin–orbit doublet appearing at binding energies approximately 1 eV lower than those characteristic of a Ti^4+^ spin–orbit doublet. The symmetric character of the Ti 2p spectral lines recorded from the materials prepared in this work ruled out the presence of a Ti^3+^ contribution. In conclusion, our low temperature method produced a CTO with only Ti^4+^, in contrast with results recently reported for CTO prepared by a high-temperature solid-state reaction, where Ti presented a trivalent character in this compound [64].

The O 1s spectra (Figure 3c) contain three contributions. The main one (approximately 75%) at 530.1 eV is characteristic of metal–oxygen bonds and has to be associated with the Co-O and Ti-O bonds existing in Co_2_TiO_4_ [63,65]. The contribution at 532.3 eV can be assigned to C=O bonds or an array of physi- and chemisorbed water molecules on the surface of the CTO particles. The small contribution (less than 5%) at approximately 535.0 eV is likely due to some unspecific organic moiety.

As reported previously, the difference between the O 1s and Ti 2p_3/2_ binding energies is also representative of the oxidation state of titanium [69]. An energy difference between 71.2 and 72.2 eV is characteristic of Ti^4+^, while an energy difference between 72.9 and 73.4 eV is characteristic of Ti^3+^. In the case of the materials considered in this work, the energy differences observed were 71.6 eV (CTO) and 71.9 eV (CTO/RGO) (i.e., both were characteristic of Ti^4+^). It has also been reported that a correlation exists between this energy difference and the Ti-O bond length. In the case of the materials examined here, the energy difference was slightly larger for the composite material, which would imply the occurrence of slightly longer Ti-O bonds that probably arose from a more distorted coordination geometry [25].

The Co/O and Ti/O atomic ratios obtained from the evaluation of the XPS data were 0.38 and 0.18 for CTO, respectively, and 0.41 and 0.15 for CTO/RGO, respectively. These Co/O and Ti/O atomic ratios, which were calculated while considering that only 75% of the oxygen corresponded to reticular oxygen, were relatively close to the nominal values of 0.5 and 0.25, respectively.

To verify the amount of carbon material present in the CTO/RGO in situ hybrid, a thermogravimetric analysis (TGA) was carried out in a temperature range between 30 and 1000 °C and in an air atmosphere (Figure S2). The steep loss observed at approximately 450 °C (20.8 wt.%) is attributed to the decomposition of the RGO present in the hybrid [57].

**Figure 4:**
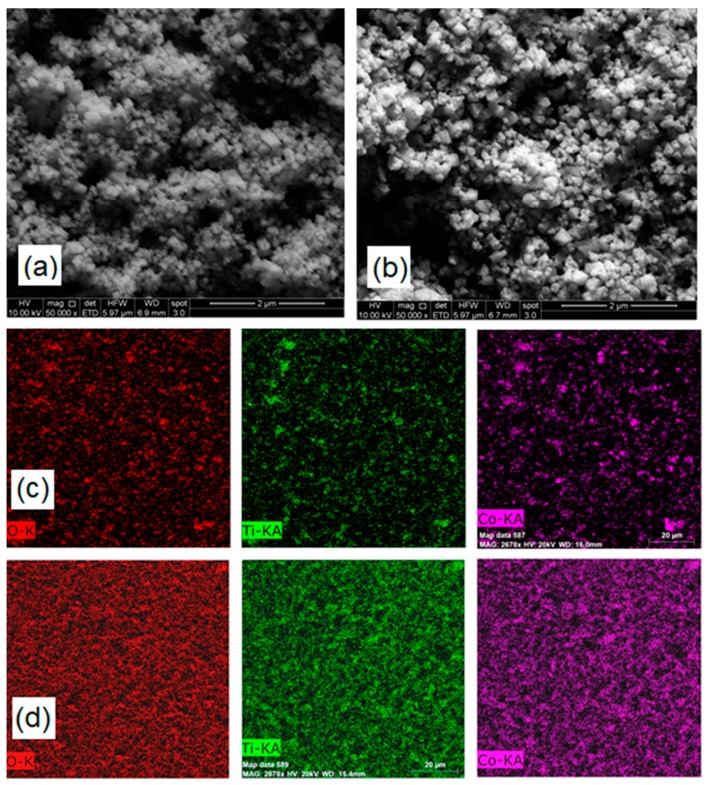
SEM micrographs of CTO+RGO (**a**) and CTO/RGO (**b**). EDX mapping (oxygen, titanium, and cobalt) of CTO+RGO (**c**) and CTO/RGO (**d**).

Particle morphologies in the hybrid materials were studied by SEM. In both hybrid materials, CTO presented a polyhedral morphology, as shown in Figure 4a,b. However, according to the histogram size distribution shown in Figure S3, both materials have average particle sizes of 103 ± 26 nm and 103 ± 43 nm for CTO/RGO and CTO+RGO, respectively. The in situ synthesis appears to produce a slightly more homogeneous size distribution of CTO nanoparticles in the hybrid material.

EDX maps, as shown in Figure 4, were used to study the distribution of Co, Ti, and O in the hybrid materials. Comparison of Figure 4c,d shows that CTO/RGO presents a more homogeneous elemental distribution than CTO+RGO. Since the CTO/RGO method involves the formation and growth of the inorganic material in the presence of the carbonaceous material at the nanometric scale, it enables more uniform distribution of elements in the polycrystalline material by controlling the nucleation sites in the nanomaterials through the functionalization of the surfaces [25].

According to the literature, the oxide nanoparticle synthesis method can affect the particle size and shape, the particle size distribution, and the degree of crystallinity [70]. In our case, the in situ hydrothermal synthesis method produced CTO particles with a narrower size distribution.

**Figure 5:**
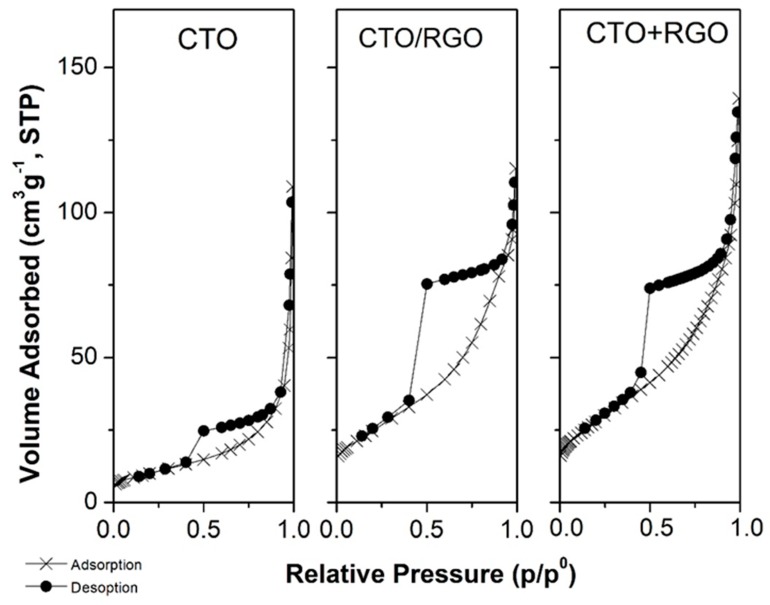
N_2_ adsorption–desorption isotherm profile.

The nitrogen sorption isotherms of CTO and both hybrid materials are shown in Figure 5. All the compounds exhibit a typical type IV behaviour with a H2(a) hysteresis loop characteristic of materials that contain mesopores [71]. The presence of RGO in the hybrids produces a large increase in the porosity compared to the CTO.

The BET specific surface area for pristine CTO synthesized by the hydrothermal method was 36 m^2^/g, higher than the value of 4 m^2^/g reported for CTO synthesized by methods with high calcination temperatures [72]. It is expected that this higher surface area obtained for hydrothermally synthetized CTO will increase the electrocatalityc performance against hydrogen peroxide determination. Table 1 shows the texture analysis corresponding to the hybrid materials. The incorporation of RGO, either in CTO+RGO or CTO/RGO, produces an increase in the BET areas (S_BET_) to 100 m^2^/g and 90 m^2^/g, respectively. Both hybrids have a similar mesoporous volume (V_m_); however, the in situ hybrid presents 100% mesoporous-type pores, without any micropore (V_0_) formation observed; the ex situ hybrid exhibits 86.3% mesopore volume (V_m_) and 13.7% micropore volume (V_0_).

**Figure 6:**
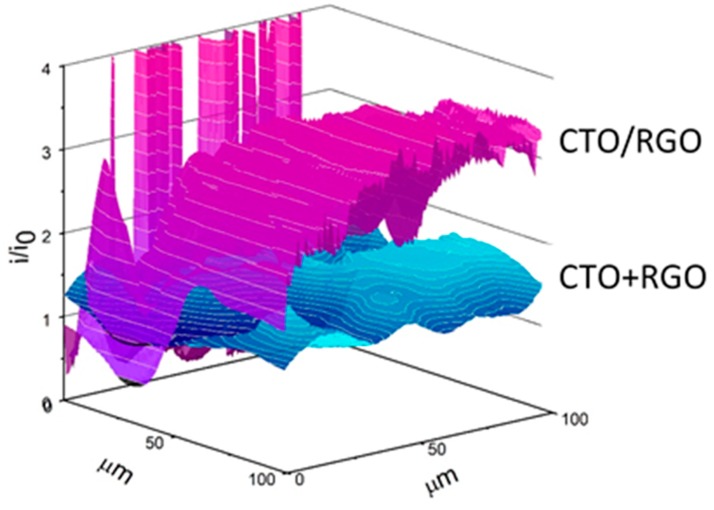
Scanning electrochemical microscopy (SECM) feedback plot for the CTO/RGO and CTO+RGO electrodes using ferrocene methanol as the redox mediator.

To characterize both the topography and electroactivity of electrodes modified with the hybrid material, scanning electrochemical microscopy (SECM) in feedback mode was used. SECM allowed us to characterize both the topography and electroactivity of newly modified surfaces. Figure 6 displays the normalized currents (i/i0) of UME when scanning the CTO/RGO and CTO+RGO surfaces using FcOH as a redox mediator. The electrode modified with CTO/RGO showed higher normalized currents than the one modified using CTO+RGO. This current increase could be attributed to a better coupling between the components of the hybrid product of in situ synthesis and a better distribution of CTO on the graphitic material, resulting in improved electroactivity. As in previous reports [73,74], there were regions with different electroactivity, probably due to a non-homogeneous dispersion of hybrids that generate areas with dissimilar concentrations on the electrode surface.

### 3.2. Electrochemical Behaviour of Glassy Carbon Electrodes Modified with the Hybrid Materials towards Hydrogen Peroxide

**Figure 7:**
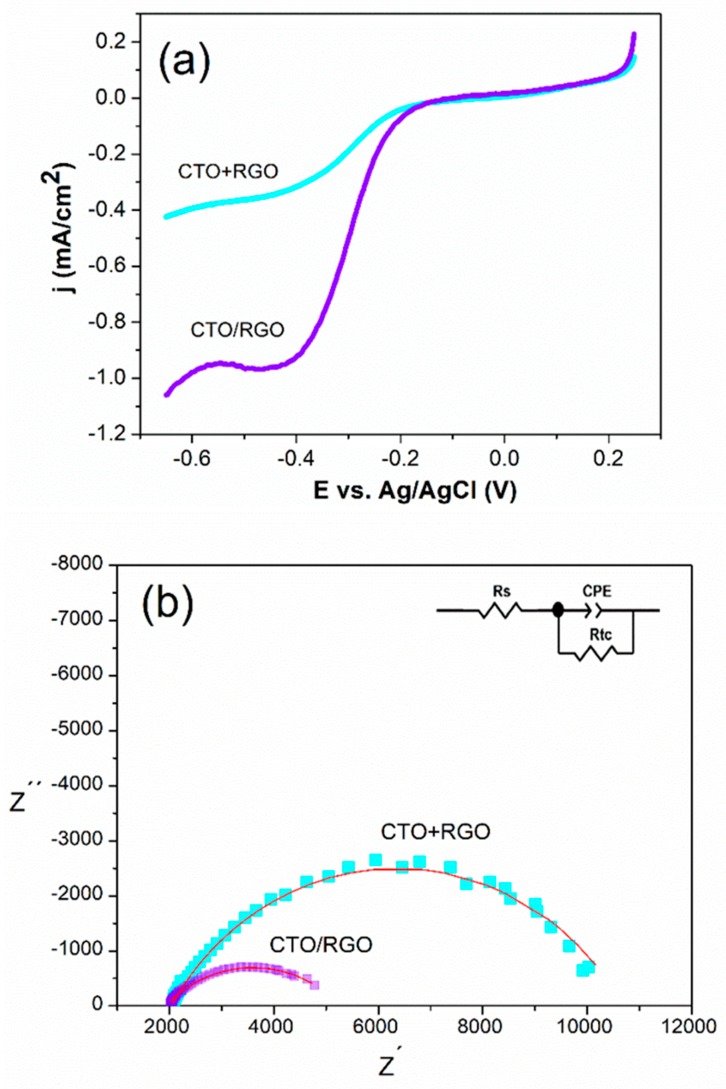
Polarization curves for reduction of 1.0 mM H_2_O_2_ on GCEs modified with CTO/RGO and CTO+RGO hybrids in N_2_-saturated pH 12 NaOH. Sweep rate of 5 mV/s and rotating speed of 1600 rpm (**a**). Nyquist plot of 1.0 mM H_2_O_2_ on CTO/RGO and CTO+RGO hybrids in pH 12 NaOH at −0.400 V. Inset on figure: Equivalent circuit. (**b**) The symbols represent the experimental results, and the solid line represents the corresponding fit with the equivalent circuit.

The electrocatalytic activity of the hybrid materials towards hydrogen peroxide was studied using a GCE modified with nanohybrids dispersed in Nafion^®^. Figure 7a shows the polarization curves for the reduction of hydrogen peroxide with GCEs modified with CTO+RGO and CTO/RGO. The onset potentials for the reduction of hydrogen peroxide at CTO/RGO and CTO+RGO were −0.180 V and −0.210 V, respectively, which were values lower than those reported for other similar hybrid materials of graphene and metallic oxides [75]. The current density obtained using CTO/RGO (0.952 mA/cm^2^) was three times higher than that obtained using CTO+RGO (0.335 mA/cm^2^). Thus, CTO/RGO exhibited better electrocatalytic activity towards hydrogen peroxide reduction. The selected working potential for further studies was −0.400 V, as this gave a sensitive and stable signal for the reduction of hydrogen peroxide.

To obtain additional information about the behaviour of the nanohybrid-modified electrodes, we performed electrochemical impedance spectroscopy experiments at −0.400 V, using hydrogen peroxide as the redox probe. Figure 7b shows the Nyquist plots obtained for the modified CTO/RGO and CTO+RGO glassy carbon electrodes. The equivalent circuit is (R_s_(R_ct_CPE)) (displayed in the inset of Figure 7b), where R_s_ is the resistance of the solution, R_ct_ is the charge transfer resistance, and CPE is a constant phase element. The impedance is defined by Equation (1):

(1) $$Z_{\mathrm{CPE}}\;=\;\frac{1}{{\left(j\omega \right)}^{n}C}$$

where C is the capacitance and n is the parameter that indicates the behaviour of the CPE, varying between 1 ≥ n ≥ 0 [76]. Table 2 summarizes the different EIS parameters. It was determined that the electroactive area values for the CTO/RGO and CTO+RGO hybrids were similar (0.044 ± 0.003 cm^2^ and 0.045 ± 0.004, respectively), however, there were differences in the capacitance values, indicating that there was no relationship with the number of active sites [77,78].

A higher capacitance value for the CTO/RGO hybrid was related to an increased coupling between both nanomaterials; this increased interaction facilitated the transfer of charge and improved the electrochemical activity, which is reflected in the lower resistance (R_ct_).

### 3.3. Amperometric Detection of Hydrogen Peroxide

**Figure 8:**
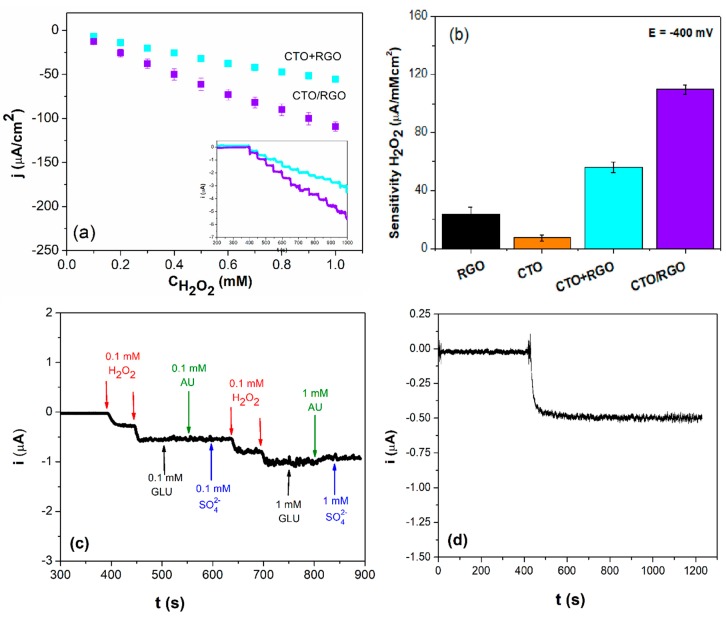
Current density vs. H_2_O_2_ concentration plot, obtained from the amperometric experiment (**a**). Amperograms of CTO/RGO and CTO+RGO obtained after ten 0.1 mM H_2_O_2_ additions (insert figure (a). Sensitivity calculated for CTO/RGO, CTO+RGO, RGO, and CTO (**b**). Amperometric responses of the CTO/RGO to successive additions of H_2_O_2_, uric acid (AU), glucose (GLU), and sodium sulfate (**c**). Stability of the response to 1.0 mM H_2_O_2_ for CTO/RGO (**d**) at −0.400 V in pH 12 NaOH.

Figure 8a depicts the calibration plots obtained for CTO/RGO and CTO+RGO from amperometric experiments at −0.400 V with additions of 1.0 × 10^−4^ M hydrogen peroxide, as shown in the inset. A clear and well-defined response was obtained with both electrodes, with a more sensitive response from the CTO/RGO electrode (110 ± 3 µA/mMcm^2^) than that for the CTO+RGO electrode (54 ± 4 µA/mMcm^2^). The detection limits (taken as 3.3 σ/S, where σ is the standard deviation of the blank signal and S is the sensitivity) were 2.1 × 10^−6^ M and 1.1 × 10^−6^ M for CTO/RGO and CTO+RGO, respectively. The reproducibility values obtained for the different electrodes were 2.9% for CTO/RGO and 6.1% for CTO+RGO.

The strong coupling between CTO and RGO in the CTO/RGO hybrid can improve the dispersion of CTO nanoparticles on the surfaces of RGO during the synthetic procedure, decreasing the resistance and increasing the electrical conductivity of the final material. Also, the higher mesoporosity of the CTO/RGO hybrid allows the easy access of H_2_O_2_ to active sites, as well as the accessibility of the electrolyte.

A comparative bar plot for the sensitivities obtained using GCEs modified with both materials separately (i.e., RGO, CTO, and with the hybrids CTO+RGO and CTO/RGO) is shown in Figure 8b. The results show that there is a clear synergistic effect when CTO and RGO are present as nanohybrid materials, either in the case of CTO/RGO or CTO+RGO, although the GCE modified with the nanohybrid material prepared in situ produces the highest sensitivity as a result of the intimate contact between the individual components. As the RGO was evaluated itself as an electrocatalyst, it is possible to conclude that the impurities reported for this material (see Supplementary Materials) do not influence the synergist effect of the hybrid materials.

A comparison of our results with other reported analytical sensors based on RGO/metallic oxide composites or cobalt oxide alone is present in Table 3. As can be seen, our method presents Limit of Detection (LOD) comparable to those obtained with other methods, with a high sensitivity achieved using the lower applied potential (i.e., is more efficient than the other reported methods).

Given that CTO/RGO showed better sensitivity, reproducibility, and a greater synergistic effect than that of the CTO+RGO, the selectivity and stability was evaluated with CTO/RGO-modified GCE.

Figure 8c displays the amperometric response of hydrogen peroxide for CTO/RGO at −0.400 V after two additions of 1.0 × 10^−4^ M hydrogen peroxide and successive additions of glucose, uric acid (representing biological interferents), and sulfate (cleaning product interferent). The CTO/RGO platform generated a negligible response in the presence of interferents, indicating a high selectivity for H_2_O_2_ detection. Figure 8d shows the short-term stability of the amperometric response to 1.0 × 10^−4^ M hydrogen peroxide recorded over a continuous time period of 800 s. The response of the CTO/RGO electrode remained stable throughout the entire experiment, with only a 4.6% decrease in current. Finally, the response time of the surface after the addition of hydrogen peroxide was fast, taking no more than 10 s to establish a stable current.

Furthermore, to verify the applicability of the CTO/RGO hybrid material, H_2_O_2_ detection was performed using a real sample (a commercial laundry whitening product, Vanish^®^). The results showed that our amperometric method detected a H_2_O_2_ concentration value of (1.900 ± 0.004) M, and the reference method (titration method) detected a concentration of (1.520 ± 0.060) M. Both values were above the value declared by the manufacturer (1.25 M) but demonstrated future analytical usefulness for determining hydrogen peroxide content in real samples after an adequate treatment of the samples or the incorporation of an anti-interferents membrane on the resulting electrodes.

## 4. Conclusions

For the first time, the synthesis of nanostructured CTO at low temperature, alone and in the presence of RGO, and using a one-step hydrothermal synthesis method with new precursors and low temperatures is reported. The CTO/RGO and CTO+RGO hybrids demonstrated electrocatalytic activity towards the electroreduction of hydrogen peroxide. More importantly, a better coupling between CTO and RGO during the in situ synthesis process promoted an effective GCE/CTO/RGO electrode, which was shown to be an interesting and simple alternative for the quantification of hydrogen peroxide and which opens doors for further electrochemical sensor development.

## Figures and Tables

**Table 1:** Textural analysis for CTO/RGO and CTO+RGO.

Hybrid Material	V_0_ (cm^3^/g)	V_m_	S_BET_ (m^2^/g)
CTO+RGO	0.029	0.182	100
CTO/RGO	0.000	0.173	90

**Table 2:** EIS parameters obtained from the Nyquist plots shown in Figure 7.

Hybrid Material	C (10^−5^ F)	n	R_tc_ (Ω)
CTO+RGO	1.6 ± 0.1	0.68 ± 0.02	8422 ± 1591
CTO/RGO	8.4 ± 1.9	0.51 ± 0.01	3679 ± 287

**Table 3:** Comparison of the analytical performance of various electrodes for H_2_O_2_ sensing.

Electrode	Electrolyte	Potential (V)	Sensitivity (μAmM^−1^cm^−2^)	LOD (µM)	Ref.
MnO_2_-Co_3_O_4_/RGO	PBS (pH = 7.4)	+0.50	53.6	0.8	[79]
Co_3_O_4_	NaH_2_PO_4_-NaOH (pH 10)	−0.70	-	4.4	[80]
Pt/Fe_3_O_4_/RGO	PBS (pH = 7.4)	0.00	6.56	1.56	[81]
NiCo_2_S_4_/RGO	NaOH (pH13)	−0.45	118.5	0.19	[82]
MnO_2_/RGO	PBS (pH = 7.4)	−0.50	59.0	10	[83]
CTO/RGO-25	NaOH (pH12)	−0.40	106	2.1	(*)

(*) This work.

## Supplementary Materials

The following are available online at https://www.mdpi.com/2079-4991/9/11/1611/s1, Figure S1: Raman frecuency shift for RGO in CTO/RGO hybrid, Figure S2: TGA curve of CTO/RGO, Figure S3: Histogram particle size distribution of CTO. Table S1: Elemental Analysis of RGO, supported by Graphenea, Table S2: X-ray fluorescence spectrometry analysis of RGO, supported by Graphenea, Table S3: Rietveld Refinement results summary.

## Abbreviations

CTO, Co_2_TiO_4_; RGO, reduced oxide graphene; NPs, nanoparticles; CBNM, carbon-based nanomaterials.
