# Supplementary material for: Co2TiO4/Reduced Graphene Oxide Nanohybrids for Electrochemical Sensing Applications
Source: Nanomaterials (Basel). 2019 Nov 13;9(11):1611. doi: 10.3390/nano9111611 (PMC6915725; doi:10.3390/nano9111611)
Supplement: Supplementary file 1 [file nanomaterials-09-01611-s001.pdf]

Appendice

Co_2TiO_4/reduced graphene oxide nanohybrids for electrochemical sensing applications

Constanza J. Venegas[1,2], Fabiana A. Gutierrez[3], Marcos Eguílaz[3], José F. Marco[4], Nik Reeves-McLaren[5], Gustavo A. Rivas[3*], Domingo Ruiz-León[2*] and Soledad Bollo[1,6*]

Table S1. Elemental Analysis of RGO, supported by Graphenea:.

	RGO %
C	77–87%
H	0–1%
N	0–1%
S	0
O	13–22%

Table S2. X-ray fluorescence spectrometry analysis of RGO, supported by Graphenea:.

**Datum**	**17.07.17**
**Bemerkung**	11987-1 rGO-1 v. Graphenea
**Methode [ppm]**	FP_10_Graphit 99
**Na**	695
**Mg**	3.4
**Al**	1.5
**Si**	225.6
**P**	3.6
**S**	432.8
**Cl**	8.1
**K**	126.7
**Ca**	150.6
**Cr**	36.8
**Mn**	3413
**Fe**	106.7
**Co**	1
**Ni**	17.9
**Cu**	0.5
**Zn**	5.2
**As**	0.2
**Br**	0.4
**Sr**	1.9
**Zr**	8.4
**Mo**	10.4

**Cd**	2.5
**Sn**	2.1
**Sb**	6.9

Table S3. Rietveld Refinement results summary.

CTO/RGO

	Mult	Fractional Coordinates (x,y,z)			Occ	Uiso
**Co_tet**	8	0.375	0.375	0.375	0.979(6)	0.0100
**Ti_tet**	8	0.375	0.375	0.375	0.021(6)	0.0100
**Co_oct**	16	0.000	0.000	0.000	0.478(3)	0.0100
**Ti_oct**	16	0.000	0.000	0.000	0.522(3)	0.0100
**O**	32	0.240	0.240	0.240	1.000	0.0090

Lattice parameter: a = 8.4519(1) Å, X^2 = 3.779, wRp = 2.33%, Rp = 3.75%

CTO+RGO

	Mult	Fractional Coordinates (x,y,z)			Occ	Uiso
**Co_tet**	8	0.375	0.375	0.375	0.959(7)	0.0100
**Ti_tet**	8	0.375	0.375	0.375	0.041(7)	0.0100
**Co_oct**	16	0.000	0.000	0.000	0.490(3)	0.0100
**Ti_oct**	16	0.000	0.000	0.000	0.510(3)	0.0100
**O**	32	0.239	0.239	0.239	1.000	0.0092(2)

Lattice parameter: a = 8.4554(1) Å, X^2 = 4.474, wRp = 2.54%, Rp = 3.86%

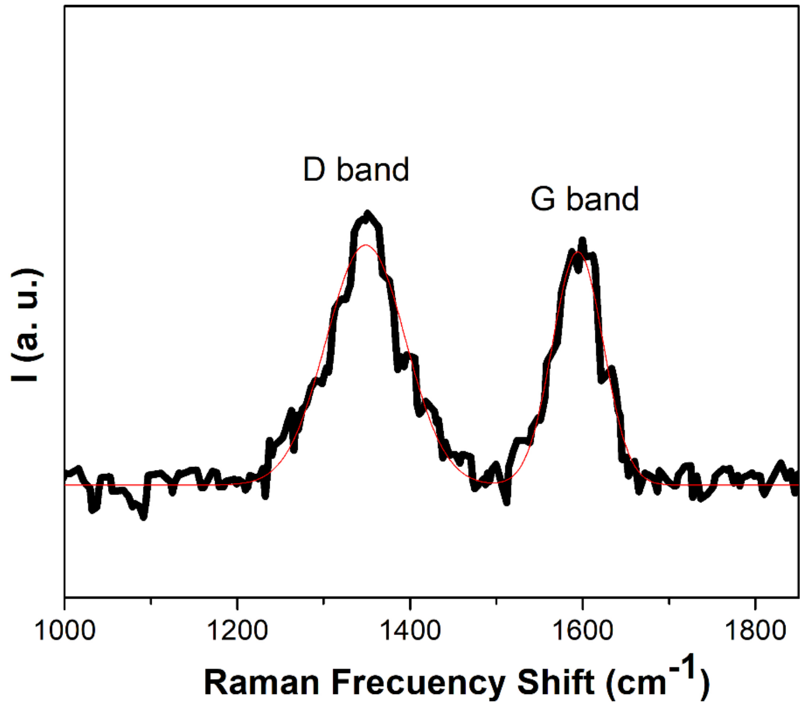

Figure S1. Raman frecuency shift for RGO in CTO/RGO hybrid.

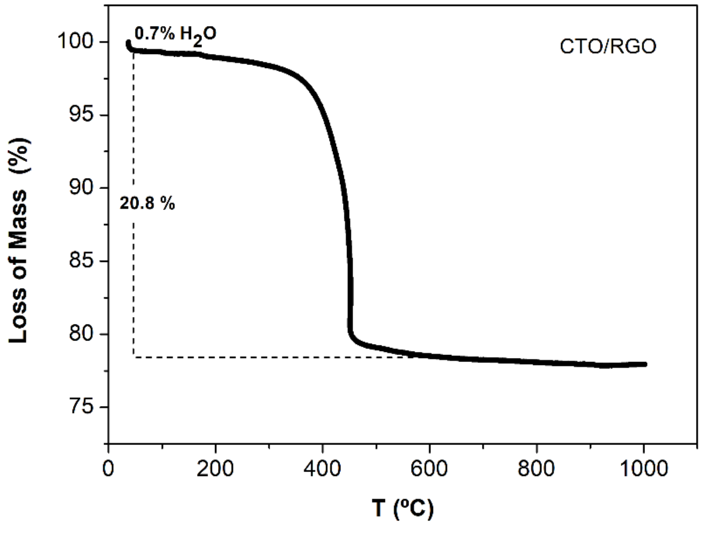

Figure S2. TGA curve of CTO/RGO.

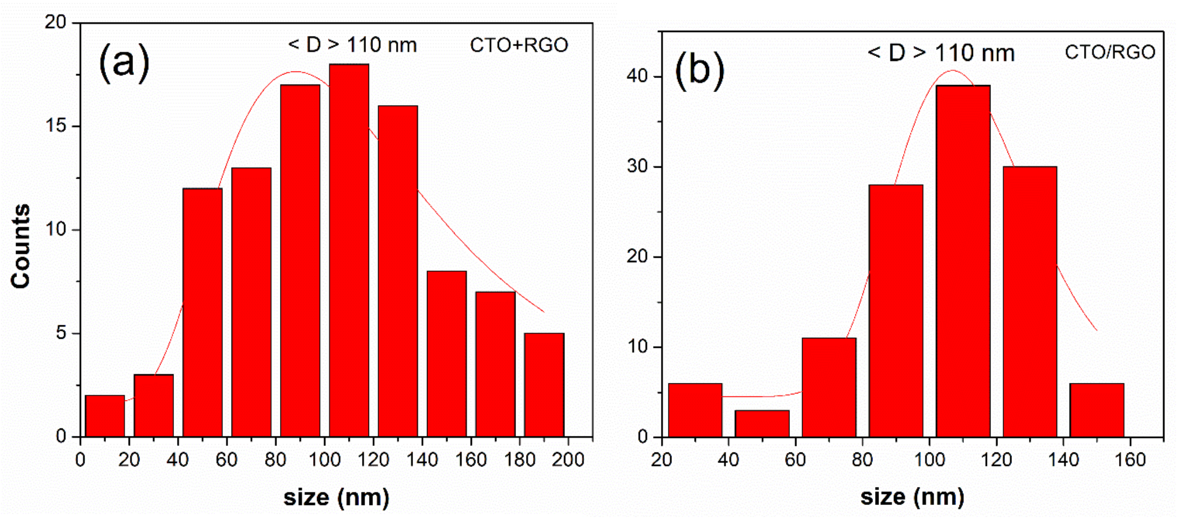

Figure S3. Histogram particle size distribution of CTO in, CTO+RGO (a), CTO/RGO (b).

CTO_OGR_2

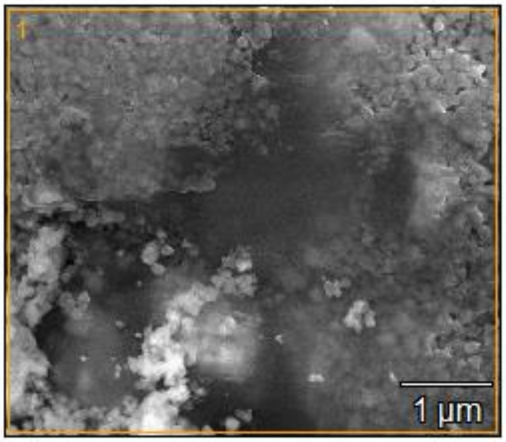

Image Name: CTO_OGR_2
Image Resolution: 512 by 444
Image Pixel Size: 0.01 µm
Acc. Voltage: 20.0 kV
Magnification: 49933

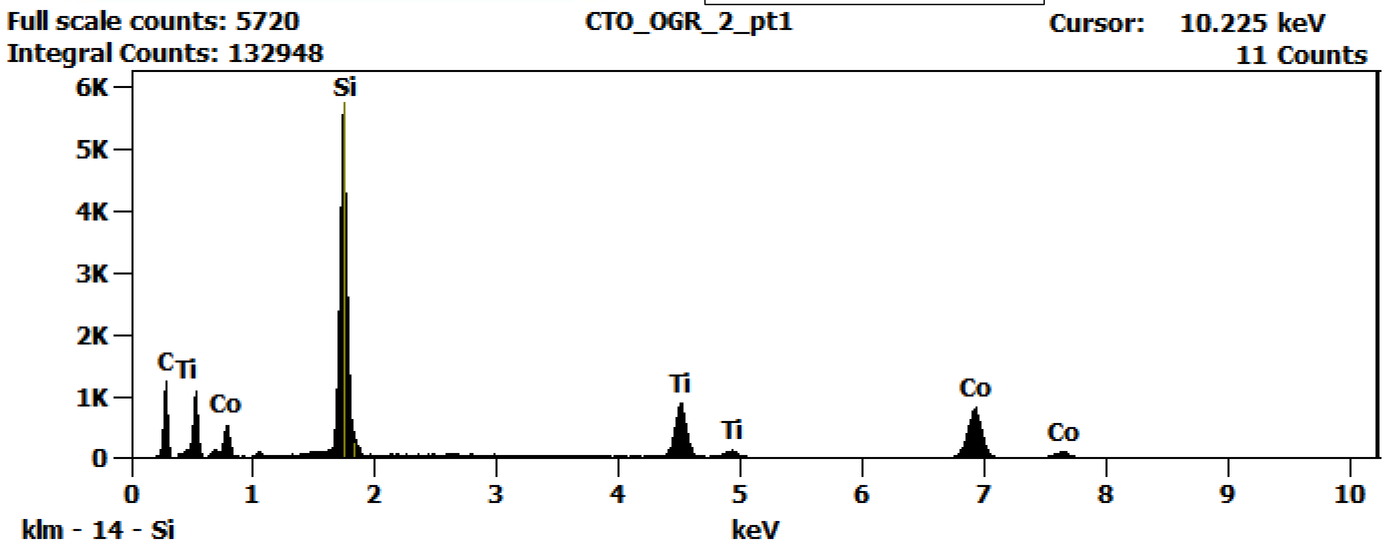

Element	CTO_OGR_2_pt1	CTO_OGR_2_pt1	CTO_OGR_2_pt1	CTO_OGR_2_pt1	CTO_OGR_2_pt1	CTO_OGR_2_pt1	CTO_OGR_2_pt1
	Net Counts	Net Counts err	Weight %	Weight % err	Atom %	Compound %	Standard Name
**Ti K**	11726	170	29.69	0.43	34.19	29.69	___
**Co K**	13400	217	70.31	1.14	65.81	70.31	___
			100.00		100.00	100.00	

CTO

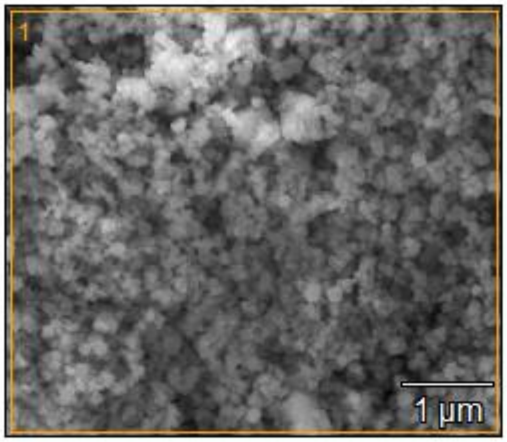

Image Name:	CTO
Image Resolution:	512 by 444
Image Pixel Size:	0.01 µm
Acc. Voltage:	20.0 kV
Magnification:	49933

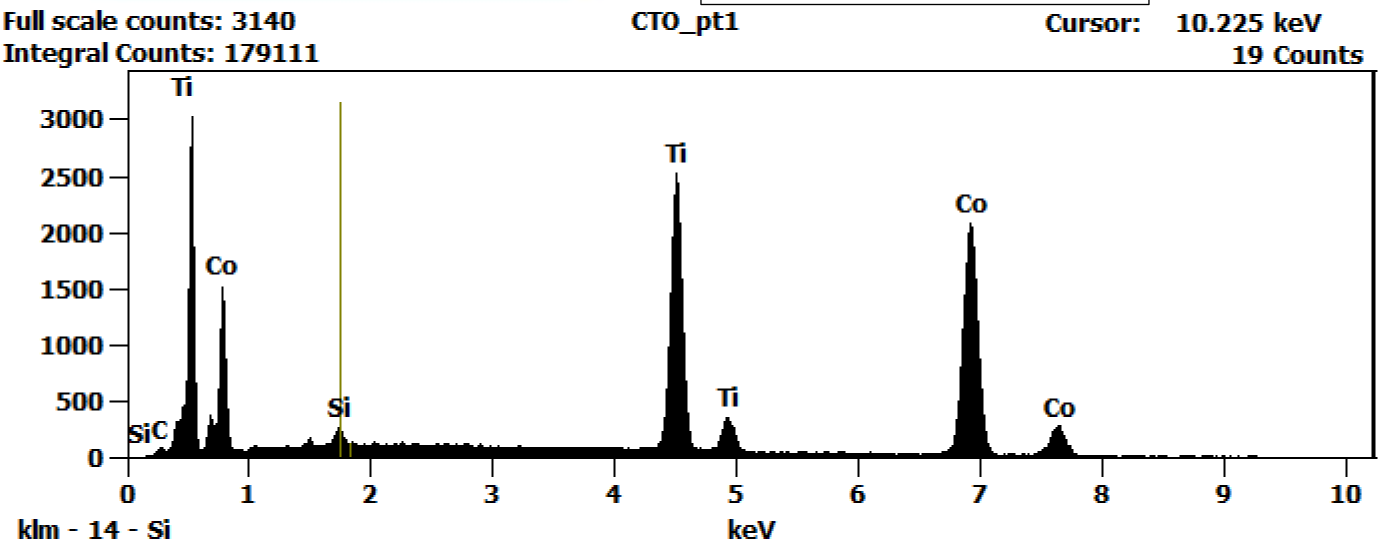

Element	CTO_pt1	CTO_pt1	CTO_pt1	CTO_pt1	CTO_pt1	CTO_pt1	CTO_pt1
	Net Counts	Net Counts err	Weight %	Weight % err	Atom %	Compound %	Standard Name
Ti K	33465	282	31.35	0.26	35.98	31.35	___
Co K	35305	336	68.65	0.65	64.02	68.65	___
			100.00		100.00	100.00	

RGO

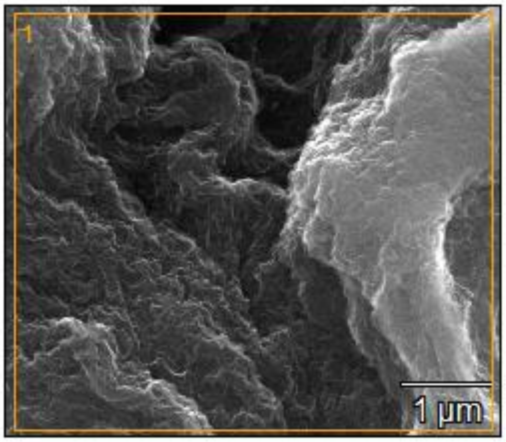

Image Name: RGO
Image Resolution: 512 by 444
Image Pixel Size: 0.01 µm
Acc. Voltage: 20.0 kV
Magnification: 49933

Full scale counts: 6146
Integral Counts: 42831

RGO_pt1

Cursor: 10.240 keV
3 Counts

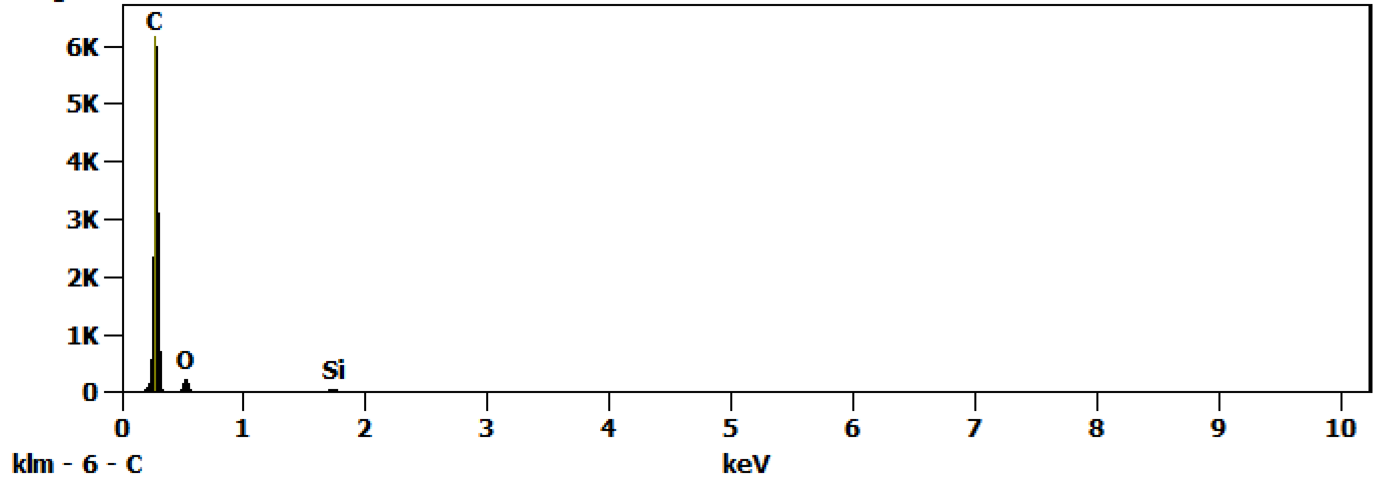

Element	RGO_pt1	RGO_pt1	RGO_pt1	RGO_pt1	RGO_pt1	RGO_pt1	RGO_pt1
	Net Counts	Net Counts err	Weight %	Weight % err	Atom %	Compound %	Standard Name
C K	29716	176	77.00	0.46	81.68	77.00	___
O K	1191	47	23.00	0.91	18.32	23.00	___
			100.00		100.00	100.00	

nanomaterials

MDPI

RGO_1

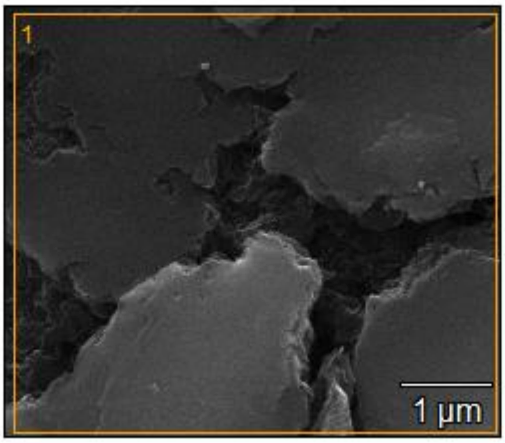

Image Name: RGO_1
Image Resolution: 512 by 444
Image Pixel Size: 0.01 µm
Acc. Voltage: 20.0 kV
Magnification: 49933

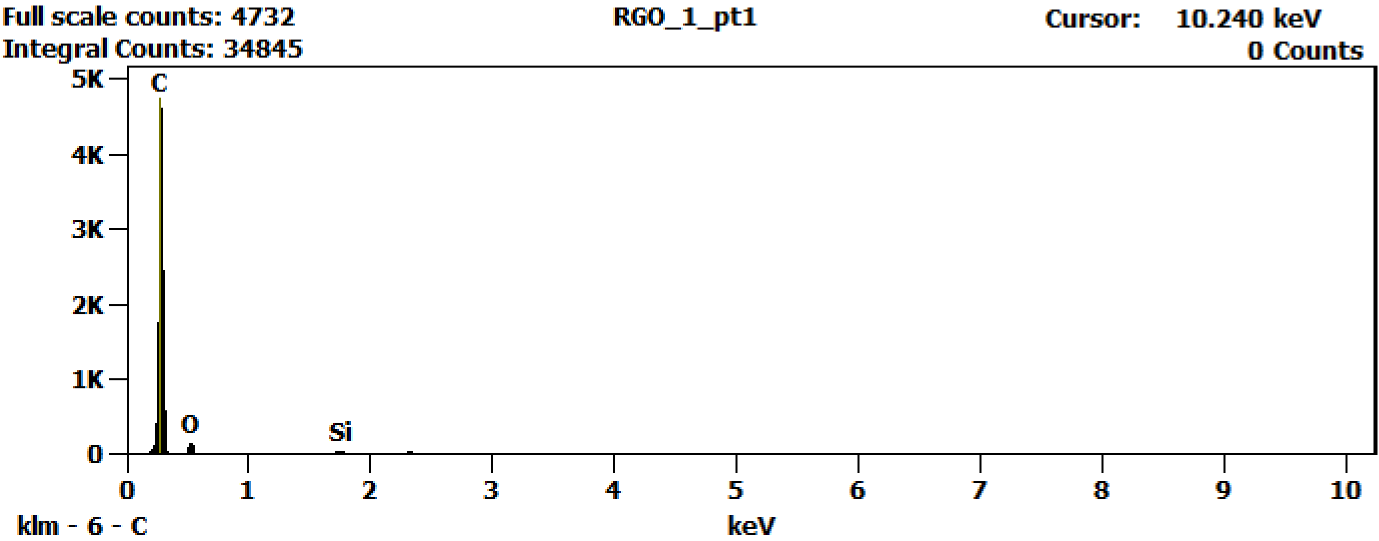

Element	RGO_1_pt1	RGO_1_pt1	RGO_1_pt1	RGO_1_pt1	RGO_1_pt1	RGO_1_pt1	RGO_1_pt1
	Net Counts	Net Counts err	Weight %	Weight % err	Atom %	Compound %	Standard Name
**C K**	22897	144	78.90	0.50	83.28	78.90	___
**O K**	783	38	21.10	1.02	16.72	21.10	___
			100.00		100.00	100.00	
